# Effects of weaning age and housing conditions on phenotypic differences in mice

**DOI:** 10.1038/s41598-020-68549-3

**Published:** 2020-07-15

**Authors:** Jeremy D. Bailoo, Bernhard Voelkl, Justin Varholick, Janja Novak, Eimear Murphy, Marianna Rosso, Rupert Palme, Hanno Würbel

**Affiliations:** 1 Division of Animal Welfare, University of Bern, Bern, Switzerland; 2 Department of Cell Biology and Biochemistry, School of Medicine, Texas Tech Health Sciences Center, Lubbock, TX USA; 3 Department of Civil, Environmental, and Construction Engineering, Texas Tech University, Lubbock, TX USA; 4 Department of Biology and UF Genetics Institute, University of Florida, Gainesville, USA; 5 Department of Behavioural Biology, University of Münster, Münster, Germany; 6 Department of Biomedical Sciences, University of Veterinary Medicine Vienna, Vienna, Austria

**Keywords:** Neuroscience, Ecology

## Abstract

Poor reproducibility is considered a serious problem in laboratory animal research, with important scientific, economic, and ethical implications. One possible source of conflicting findings in laboratory animal research are environmental differences between animal facilities combined with rigorous environmental standardization within studies. Due to phenotypic plasticity, study-specific differences in environmental conditions during development can induce differences in the animals’ responsiveness to experimental treatments, thereby contributing to poor reproducibility of experimental results. Here, we studied how variation in weaning age (14–30 days) and housing conditions (single versus group housing) affects the phenotype of SWISS mice as measured by a range of behavioral and physiological outcome variables. Weaning age, housing conditions, and their interaction had little effect on the development of stereotypies, as well as on body weight, glucocorticoid metabolite concentrations, and behavior in the elevated plus-maze and open field test. These results are surprising and partly in conflict with previously published findings, especially with respect to the effects of early weaning. Our results thus question the external validity of previous findings and call for further research to identify the sources of variation between replicate studies and study designs that produce robust and reproducible experimental results.

## Introduction

Over the last years, concerns have been raised that reproducibility of experimental results in laboratory animal research may be at stake^1–3^. These concerns may in part be due to the limited external validity of individual studies^4–6^, which in combination with binary criteria of statistical significance and publication bias may produce conflicting findings^7^. Both, experimental studies and meta-analyses suggest that the laboratory environment is an important factor contributing to variation in behavioral and physiological measures between studies^5,6,8–12^. Differences in environmental parameters between laboratories might affect animals during testing (through variation in test paradigms), or they might produce variation in the animals’ phenotypes (through variation in early experiences and housing conditions). If common variation in early experiences and housing conditions shapes the animals' phenotypes in such a way that it substantially affects behavioral and physiological outcome variables, then phenotypic plasticity can be an important factor contributing to conflicting findings in laboratory animal research^13^.

Two aspects of early experience and housing conditions that commonly vary between studies on laboratory mice are weaning age and single versus group housing. Weaning, the detachment of the offspring from the mother, as part of the transition to nutritional and behavioral independence, represents a major challenge during early development. It is, therefore, not surprising that weaning plays an important role in shaping the development of various structural, neurological, physiological, and behavioral traits^14–17^. Moreover, the disruption of the mother–offspring relationship prior to weaning has been shown to have persistent effects on offspring phenotype in mice and rats^18–22^.

Under naturalistic conditions, physiological and behavioral changes related to weaning in mice occur between postnatal days 14 and 17, with decreases in food intake and pup-directed behavior by the dam, and a transition to the eating of solid food by the pups^23–25^. Weaning typically ends around postnatal day 23^23–25^, but depending on the litter size can extend until postnatal day 30–35^26–29^.

In commercial breeding facilities, weaning occurs usually in the third week after birth. If dams are checked for parturition twice weekly—which according to personal communication with various commercial breeders seems to be common practice—newly weaned animals may range in age from 17 to 25 days of age. In addition to the timing of weaning, the process of weaning itself differs between the natural condition and the routine in breeding facilities. Under natural conditions, weaning is a gradual transitionary process occurring over several days to weeks, while weaning in breeding facilities is characterized by the instant and permanent separation of the offspring from the mother at a pre-set day; usually around postnatal day 21^29^.

Due to its potential effects on the adult phenotype, including its contribution to variability in behavioral and neurophysiological tests, the effects of early weaning have been the subject of several studies. In those studies, the response of early weaned animals—where early usually refers to weaning between postnatal day 14 and 17—was contrasted to responses of standard weaned mice—i.e., mice weaned around day 21^30^. Early weaning has been reported to lead to concomitant increases in measures of anxiety and aggressive behavior^18,31–33^, an increased incidence of stereotypic behavior^34,35^, and higher hypothalamic–pituitary–adrenal (HPA) axis reactivity in response to novelty and stress^18^. Additionally, early weaned male mice showed neurological and neurophysiological changes, including decreased BDNF levels in the prefrontal cortex and hippocampus^36^, precocious myelination in the amygdala, and reduced bromodeoxyuridine immunoreactivity in the dentate gyrus^31^. At the proximate level, these changes seem to be mediated by the consequential elevation of corticosterone levels at and subsequent to weaning^18,36^. Finally, recent research has also provided evidence for epigenetic changes in the germline at promoter sites of several candidate genes associated with early weaning^37^.

Few studies have investigated the effects of late weaning (> 21 days of age), but there is some evidence that the effects of late weaning are opposite to early weaning—leading to reduced anxiety and more social behavior^27,28^. Terranova and colleagues studied the effect of weaning age and its consequences for mouse social and non-social behavior when treated with a delta-opioid antagonist, SNC80^14^. For this purpose, they weaned SWISS CD-1 mice at 15, 20, and 25 days. Importantly, they found treatment × sex × weaning age interactions, with all three weaning ages having different effects on some of the outcome measures. Together with observational evidence of maternal mouse behavior under naturalistic or semi-naturalistic conditions, the results of the existing literature indicate that weaning age may interact with other aspects of the early environment to exert complex effects on the developing animal.

Single housing as opposed to group housing has also been reported to affect the phenotype of mice^38,39^. Numerous studies found that singly housed mice have alterations in corticosterone levels, neurochemistry, metabolism, growth, reproduction and dopaminergic hyperactivity in shared neural regions implicated with the performance of stereotypy^21,34,35,40–45^. Additionally, several studies comparing group housed and singly housed male rodents suggest that single housing leads to diminished coping with stressors, immunodeficiency, and a higher incidence of pathology^46^.

Given the sensitivity of the effect of single housing to life history characteristics, we hypothesized that weaning age should modulate phenotypic responsivity to housing conditions. In this study, we therefore investigated how weaning age, varied across five ages (14, 18, 22, 26 and 30 days), and housing conditions (single versus group housing) affected the phenotype of male and female mice.

## Methods

### Design

This study employed a 5 (weaning age) × 2 (housing condition) × 2 (sex) full factorial design (Table 1). The unit of measurement was a focal animal within each cage.

**Table 1:** Distribution of subjects by treatment and sex.

Weaning age	Group housing		Single housing	
	Males	Females	Males	Females
14	6 (12)	6 (12)	6	6
18	6 (12)	6 (12)	6	6
22	6 (12)	6 (12)	6	6
26	6 (12)	6 (12)	6	6
30	6 (12)	6 (12)	6	6
Total	30 (60)	30 (60)	30	30

Numbers in brackets represent non-focal animals in the group housed condition.

### Subjects

#### Dams: ordering, delivery, sorting and allocation to treatment

Two batches, each containing 22 primiparous RjORL:SWISS (hereafter SWISS) dams in their second week of gestation, were ordered from Janvier Labs, France. Batch 2 was delivered 14 weeks after batch 1. Each batch of mice was delivered in 2 boxes, 11 dams per box. All dams gave birth within 5–7 days after arrival to the laboratory and successfully reared pups. Litter sizes ranged from 9–18 pups.

The number of pups born to each litter was counted, and the sex of each pup ascertained 7–10 days after birth. Fifteen of the 22 litters per batch were selected based on the criteria that the number of males and females per litter was greater than four and where the sex ratio was ≥ 0.5 and ≤ 2.5. These fifteen litters were then allocated to weaning treatments using stratified random sampling of sex ratio and litter size (see Supplementary Table 1). A subset of the remaining litters was used to generate stimulus animals for use in the social odor test (see “Anxiety in the elevated plus-maze, open field, and social odor tests”; 15 males and 15 females per batch); the remaining animals were euthanized.

#### Pups: weaning, monitoring and isolation housing

**Figure 1:**
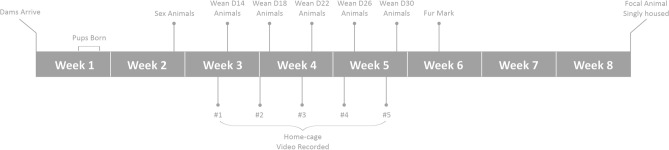
Experimental timeline until isolation housing of focal animal.

Litters were weaned according to their treatment allocation and pups were housed in same-sex groups of four animals per cage. Pups were considered for inclusion at weaning if their weight was within one standard deviation of the mean litter weight—experimental subjects were randomly selected from this pool of animals within litter. At weaning, pups were assigned a permanent intradermal ear-tattoo for identification and were given food pellets within the cage twice daily. Additionally, in experimental week 6, all animals were assigned a unique fur mark using a non-toxic animal paint marker (Stoelting Co, Illinois, USA) for individual identification during video recording (see Fig. 1). During routine weekly husbandry, one randomly selected focal animal, between ages 49–52, was removed from each cage of four animals and singly housed for the remainder of the experiment (Fig. 1). The age of single housing was selected because it is typically associated with dispersal from the natal nest and sexual maturity^47–49^.

### Husbandry procedures

Animals were kept on a reversed 12:12 light/dark cycle with lights on at 19:00. Red light emitting diodes (LED) remained on throughout the entire cycle. Temperature was maintained at 22 ± 1 °C with an average humidity of 40%. All animals were provided wood shavings (Lignocel^®^ select), 10 g of Sizzle Pet^®^ nesting material, and had ad libitum access to rodent chow (Kliba Nafag Switzerland, #3800 dams; #3430 weaned animals) and tap water.

Dams and weaned pups were housed in conditions above Switzerland’s minimum standards (c.f., Supplementary Table 2 for details). Dams were left undisturbed for the first 2 weeks after arrival to the laboratory to permit for acclimatization to the new environment and to enhance the chances of pup survival after parturition. Husbandry procedures were otherwise performed weekly for all animals.

### Outcome variables

**Figure 2:**
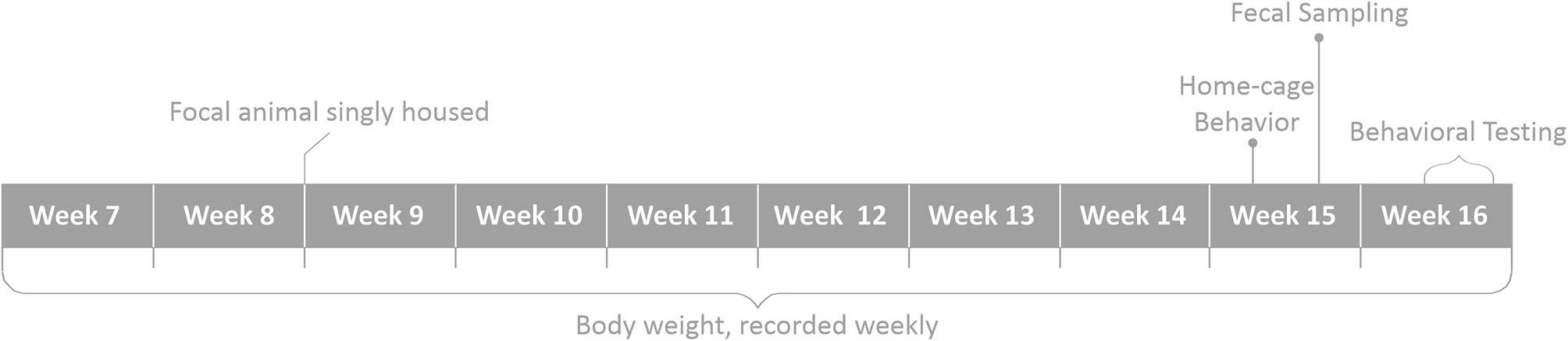
Timeline for assessment of outcome variables for a single batch of animals. Behavioral tests were conducted in the following order: elevated plus-maze, open field, and social odor test. Figure 1 indicates when maternal behavior was recorded.

Data were collected across a range of measures, including: (i) measures of behavior in the home-cage (transition to feeding from solid food in pups and stereotypy behavior performance as adults); (ii) an index of growth (body weight); (iii) indices of anxiety in three behavioral tests (elevated plus-maze behavior, open field behavior and a social odor test); and (iv) endocrine stress responses (glucocorticoid metabolites in feces) (Fig. 2).

#### Home-cage behavior

##### *Transition to feeding from solid food*

Under naturalistic conditions, weaning in mice is, in part, associated with a transition to the feeding from solid food^23–25^. Pups were therefore recorded when feeding from the food hopper using instantaneous sampling at 1-min intervals, yielding 180 data points per cage per time-point (see Supplementary Table 3 for ethogram). Each cage of mice was video recorded for 24 h prior to each weaning time-point (see Fig. 2). Mice were observed for 30 min, within 6 1-h time windows for each 24 h video recording; three time-windows within the light phase (7:00–7:30; 11:00–11:30; 15:00–15:30) and three within the dark phase (19:00–19:30; 23:00–23:30; 3:00–3:30). Video–recordings were scored using Noldus Observer XT (version 11.5) by JN and by JDB for inter-rater reliability assessment only. Five percent of all videos were rescored for assessment of intra- and inter-rater reliabilities which were high throughout^50^, with κ > 0.85.

##### *Stereotypic behavior*

We predicted that earlier weaning followed by single housing should either increase the probability of or potentiate the expression of stereotypic behavior. To investigate this prediction, all cages were recorded for 24 h each prior to the end of the experiment. Video-recordings were then scored for stereotypic behavior using Solomon coder (version 17.03.22) by JN and by MR for inter-rater reliability assessment only. For each cage, all animals were observed and the duration of stereotypic behavior scored using continuous sampling (see Supplementary Table 4 for ethogram). Mice were observed for 15 min within five 1-h time windows (09:00–10:00, 10:00–11:00, 12:00–13:00, 13:00–14:00 and 14:00–15:00). These time windows had been determined by pilot observations to represent intervals of high activity. This method yielded 75 min of coding per animal for each treatment. Ten percent of all videos were rescored for assessment of intra- and inter-rater reliabilities, which were high throughout^50^, with κ > 0.84.

#### Growth: body weight

Experimental animals were weighed at weaning and every 4 days thereafter, until 34 days of age. Subsequently, animals were weighed weekly during routine animal husbandry.

#### Anxiety in the elevated plus-maze, open field, and social odor tests

We used four elevated plus-mazes and four open field arenas; the social odor test was also performed in the open field arena. Elevated plus-mazes were made of polycarbonate with infrared (850 nm) backlit floors. Each maze consisted of four arms, each 30 cm in length and 6 cm wide with a center square measuring 6 × 6 cm. Two arms, opposite to each other, were open with a small lip around the perimeter, 0.5 cm high, while the remaining two arms were enclosed with walls 15 cm high. The open field arenas were made of polycarbonate with dimensions 45 × 45 × 45 cm with infrared (850 nm) backlit floors. The social odor test was a modification of a commonly used social interaction test, as described by Seth and File^51,52^. Instead of using a behaving conspecific, we instead used an ethologically relevant source of anxiety (soiled bedding from an unfamiliar conspecific). This modification permitted behavioral differences to be solely attributable to exposure to a novel olfactory stimulus as opposed to the dyadic interaction of two individuals. Similar to the social interaction test, we predicted that an increase in odor investigation without an increase in motor activity would be indicative of an anxiolytic effect, whereas a decrease in odor investigation with an increase in general exploration would be indicative of an anxiogenic effect.

For the three tests, mice from each cage were randomly assigned to one of two experimenters (JN and JDB). Subjects were counterbalanced across three blocks, with each block containing five animals of each sex and weaning age within a housing condition (single or group housed). Animals were tested in the same order across all three tests.

Behavior on the elevated plus-maze and the open field was assessed in single 5-min sessions, and all tests were conducted between 900 and 1,200 h on separate days. Animals were always tested first in the elevated plus-maze, and then in the open-field because behavior in the open-field was used as a baseline in the social odor test. Additionally, this order permitted some degree of habituation to the open-field arena where the social odor test was consequently performed. Animals were placed into the center of the elevated plus-maze and the open-field, with the head facing a closed arm in the former. The outcome variables of interest in the elevated plus-maze test were: (1) distance traveled and (2) time spent in the open arms. In the open field test, we measured: (1) distance traveled; (2) time spent in the center of the arena; and (3) time spent in the corners of the arena.

The social odor test was performed on the day following the open field test, which served as our baseline comparison for exploratory behavior. A plastic container (10 × 7 × 7 cm; L × W × H) containing approximately 15 g of soiled bedding from a novel same-sexed conspecific within a mesh “potpourri” bag was placed in the center of the open field arena. Three stimulus cages for each sex were used for the provision of soiled bedding and were allocated to our treatments such that soiled bedding from a single stimulus cage was presented only once to each weaning age and housing condition. Holes, 0.5 cm in diameter and spaced approximately 3 cm apart, were located on the top and sides of the plastic container to allow for diffusion of odor cues. The container occupied approximately one half of the center area of the open field and allowed for exploration within the center area of the open field from any angle, including the top of the plastic container. The outcome variables of interest were: (1) the change in distance traveled compared to the open field test and (2) time spent-in-the center where the odor stimulus was located.

For each test session, the cage to be tested was brought to the test room and the overhead lights (120 lx) were turned on. The four animals were then each removed from their cage and placed into the apparatus by the assigned experimenter. At the end of the test, the animals were replaced into the home-cage, and the cage was returned to the housing room. Between test sessions, the arenas were cleaned with 70% isopropanol. Outcome measures were scored live by Noldus EthoVision XT (version 11.5). The accuracy of video tracking was subsequently evaluated by JDB from video recordings to ensure that issues associated with automated tracking were eliminated^53^. The detection settings for tracking were selected so that both the percentage of samples in which the subject was not found and the percentage of samples skipped were less than 1% per trial.

#### Adrenocortical activity: fecal glucocorticoid metabolite analysis

Non-invasive methods of quantifying circulating levels of glucocorticoids, a primary product of the activation of the HPA stress system, are preferable to invasive methods such as blood sampling because they do not elicit a stress response^54^. In mammals, glucocorticoids are metabolized by the liver and are excreted in both urine and feces^55^. A method of analyzing these metabolized by-products has been developed and validated^55,56^ and was used as a measure of stress-induced adrenocortical activity of the mice.

Fecal boli were collected in the dark phase under red light approximately 24 h after cage changes. A minimum of 6 boli per animal per cage was collected. Samples were immediately frozen at − 20 °C and later processed blinded to experimental treatment (JN and RP) for measurement of corticosterone metabolites (ng/0.05 g feces) according to the method described by Touma et al.^57,58^. In total, 120 samples were processed.

### Statistical analyses

All statistical analyses were performed using IBM™ SPSS Statistics (version 25). For parametric models, assumptions of normally distributed errors and homogeneity of variance were examined graphically. Based on these inspections, only one transformation was necessary—the proportion of time where animals performed stereotypic behavior was cube-root transformed. Sex was analyzed separately for each model. If both sexes showed converging trends, combined probabilities were calculated. Batch was included in all models as a fixed effect—no significant differences were observed and batch is not discussed further. The full model, including all fixed effects and their interaction, was always run. p-values are presented as actual values rounded to three decimal places. Due to multiple testing, we recommend interpreting p-values against Bonferroni corrected threshold levels of α′ = 0.0125 for body weight prior to weaning, α′ = 0.0035 for body weight after weaning, α′ = 0.0041 for behavior in the elevated plus maze, α′ = 0.0027 for behavior in the open field test, α′ = 0.0025 for behavior in the social odor test and α′ = 0.0083 for glucocorticoid metabolite concentration. This correction controls for the family wise error rate associated with each outcome measure. Raw data for all outcome measures will be made available upon request.

Transition to feeding from solid food was analyzed using Kruskall–Wallis independent sample tests due to the high degree of skewness observed in the data. Data were summed across all observation intervals and expressed as proportions. Kruskall–Wallis pairwise comparisons with a Dunn–Bonferroni correction were planned to delineate the pattern of significant differences among more than two groups.

Stereotypic behavior was analyzed using the MIXED procedure. Weaning age, and housing condition (single or group) were treated as categorical fixed effects. Data were summed across all observation intervals and expressed as proportions of active time. Overall stereotypy level, rather than stereotypy type, was used as the primary outcome measure given the high degree of heterogeneity in stereotypy type that was observed.

Body weight was analyzed using the MIXED procedure and separate analyses were run before and after the single housing of a focal animal. Prior to single housing, weaning age was treated as a categorical fixed effect, with week as a continuous covariate. Subsequent to single housing, weaning age and housing condition (single or group) were treated as categorical fixed effects, with week as a continuous covariate.

Measures of anxiety—elevated plus-maze, open field behavior and the social interaction tests—were analyzed using the MIXED procedure. Weaning age, and housing condition (single or group) were treated as categorical fixed effects. For the social interaction test only, day of testing was included as a categorical fixed effect.

Fecal glucocorticoid metabolite concentrations were analyzed using the MIXED procedure. Weaning age and housing condition (single or group) were treated as categorical fixed effects.

### Ethical statement

This study was conducted in accordance with the guidelines of the Swiss Animal Welfare Ordinance (TSchV 455.1) and was approved by the Cantonal Veterinary Office in Bern, Switzerland (permit number: BE36/17).

## Results

### Transition to feeding from solid food

**Figure 3:**
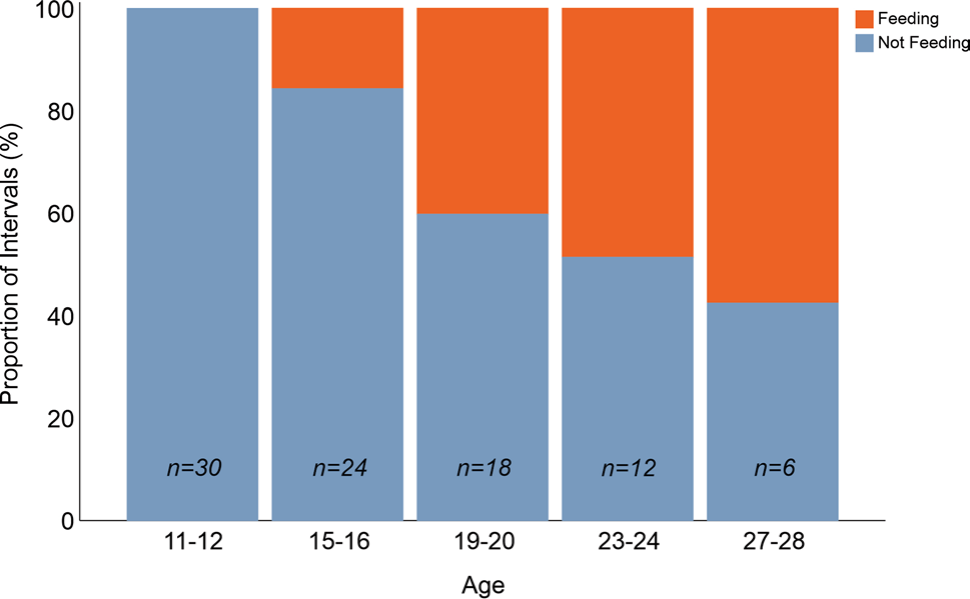
Proportion of intervals when at least one pup was observed feeding from the food hopper. The sample size, n, refers to the number of cages observed.

In general, the proportion of intervals in which prior to weaning at least one pup was observed feeding from the food hopper increased with age (*H* = 291.87, *p* < 0.001). Post hoc pairwise comparisons indicated that feeding from the hopper varied between all age groups, except between those aged 23–24 and 27–28 (Fig. 3).

### Stereotypic behavior

**Figure 4:**
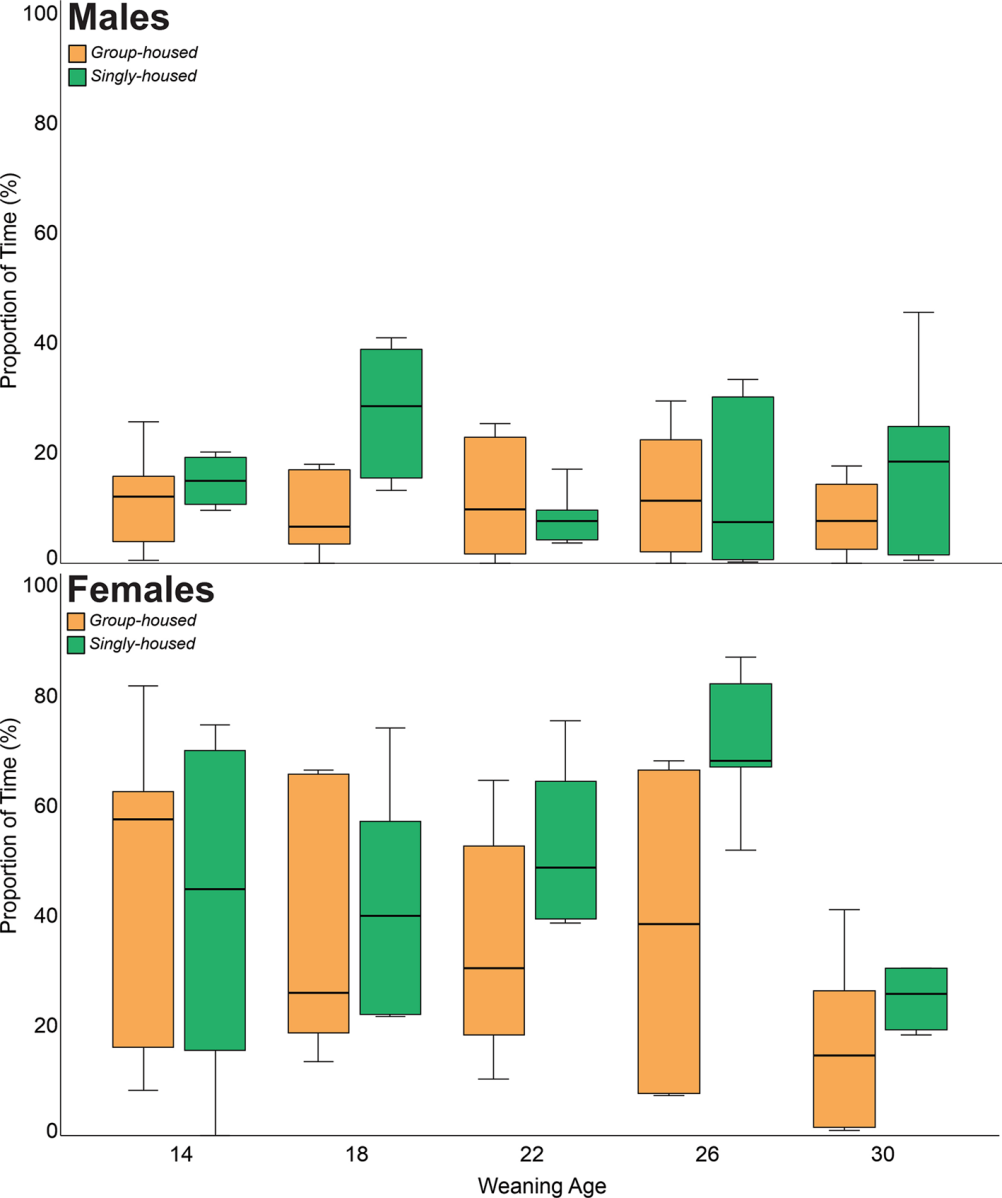
Proportion of active time (median an IQR) where animals were engaged in stereotypic behavior in relation to weaning age and housing condition.

The proportion of time in which animals performed stereotypic behavior varied only in relation to housing condition—animals that were singly housed performed more stereotypic behavior compared to those that were group housed (Fig. 4; Table 2).

**Table 2:** Summary of results by outcome measure.

Outcome variable	Sex	Weaning age	Housing condition	Time	Weaning age × housing condition	Weaning age × time	Housing condition × time	Weaning ag e × housing condition × time
**Stereotypic behavior**								
	Males	F_4,50_ = 0.912	***F***_***1,50***_*** = 4.608***	–	F_4,50_ = 2.055	–	–	–
	Females	F_1,50_ = 2.537	***F***_***4,50***_*** = 5.429***	–	F_4,50_ = 1.177	–	–	–
**Body weight**								
14–52 Days	Males	***F***_***4,409***_*** = 18.808***	–	–	–	***F***_***4,409***_*** = 13.649***	–	–
	Females	***F***_***4,409***_*** = 26.354***	–	–	–	***F***_***4,409***_*** = 20.985***	–	–
59–101 Days	Males	F_4,399_ = 0.172	F_1,399_ = 0.298	***F***_***1,399***_*** = 173.993***	F_4,399_ = 0.491	F_4,399_ = 0.158	F_1,399_ = 0.724	F_4,399_ = 0.652
	Females	F_4,400_ = 0.224	F_4,400_ = 0.299	***F***_***1,400***_*** = 131.812***	F_4,400_ = 0.396	F_4,400_ = 0.142	F_1,400_ = 2.102	F_4,400_ = 0.388
**Elevated plus maze**								
Distance traveled	Males	F_4,49_ = 1.119	*F*_*1,49*_* = 6.188*	–	*F*_*4,49*_* = 3.105*	–	–	–
	Females	F_4,50_ = 0.909	*F*_*1,49*_* = 5.805*	–	F_4,49_ = 1.173	–	–	–
Time-in-Open Arms	Males	F_4,49_ = 0.969	F_1,49_ = 3.724	–	F_4,49_ = 1.400	–	–	–
	Females	F_4,50_ = 0.566	F_1,50_ = 0.566	–	F_4,50_ = 0.479	–		
**Open-field test**								
Distance traveled	Males	*F*_*4,49*_* = 2.867*	*F*_*1,49*_* = 8.591*	–	F_4,49_ = 0.886	–	–	–
	Females	F_4,50_ = 0.676	F_1,49_ = 0.517	–	F_4,50_ = 0.354	–	–	–
Time-in-Corners	Males	F_4,49_ = 1.382	*F*_*1,49*_* = 3.798*	–	F_4,49_ = 1.384	–	–	–
	Females	F_4,50_ = 0.787	F_1,50_ = 0.225	–	F_4,50_ = 0.360	–	–	–
Time-in-Center	Males	F_4,49_ = 1.149	F_1,49_ = 4.422	–	F_4,49_ = 0.901	–	–	–
	Females	F_4,50_ = 0.482	F_1,49_ = 0.719	–	F_4,49_ = 0.183	–	–	–
**Social odor test**^a^								
Distance traveled	Males	*F*_*4,100*_* = 4.077*	***F***_***1,100***_*** = 16.241***	***F***_***1,100***_*** = 39.534***	F_4,100_ = 1.342	F_4,100_ = 1.204	F_1,100_ = 1.271	F_4,100_ = 0.407
	Females	F_4,99_ = 1.535	F_1,99_ = 1.551	***F***_***1,99***_*** = 12.784***	F_4,99_ = 0.564	F_4,99_ = 0.981	F_1,99_ = 0.011	F_4,99_ = 0.258
Time-in-Center	Males	F_4,49_ = 1.349	***F***_***1,49***_*** = 14.865***	–	F_4,49_ = 1.154	–	–	–
	Females	F_4,50_ = 0.356	***F***_***1,49***_*** = 9.757***	–	F_4,50_ = 2.244	–	–	–
**Glucocorticoid metabolites**								
	Males	F_4,50_ = 0.180	F_1,50_ = 0.336	–	F_4,50_ = 0.262	–	–	–
	Females	F_1,50_ = 0.588	F_4,50_ = 4.017	–	F_4,50_ = 1.785	–	–	–

Bolded values reflect significant differences at Bonferroni corrected thresholds for multiple testing: α′ = 0.0125 for body weight prior to weaning, α′ = 0.0035 for body weight after weaning, α′ = 0.0041 for behavior in the elevated plus maze, α′ = 0.0027 for behavior in the open field test, α′ = 0.0025 for behavior in the social odor test and α′ = 0.0083 for glucocorticoid metabolite concentration.

Cells filled in italics represent significant differences at p < 0.05 without correction for the family wise error rate.

^a^In the social odor test, the variable, time, reflects day of testing (i.e., change in comparison to the open-field test).

### Growth: body weight

**Figure 5:**
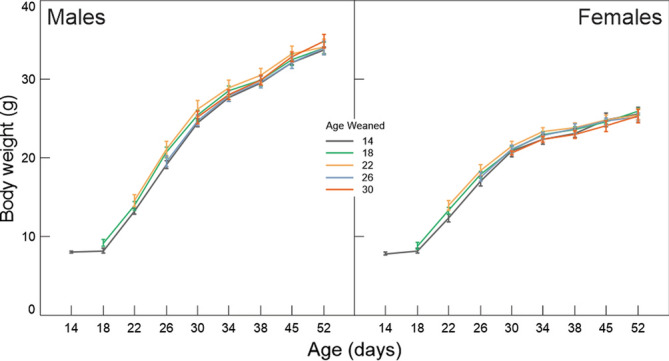
Change in body weight (mean ± SE) across time (ages 14–52) and in relation to age of weaning.

The effects of weaning age on body weight, up to 52 days of age, were restricted to animals weaned at age 14—in both male and female mice, animals weaned at 14 days of age weighed less than animals weaned at 18, 22, and 26 days of age, but only until the age of 26 days (Fig. 5; Table 2).

**Figure 6:**
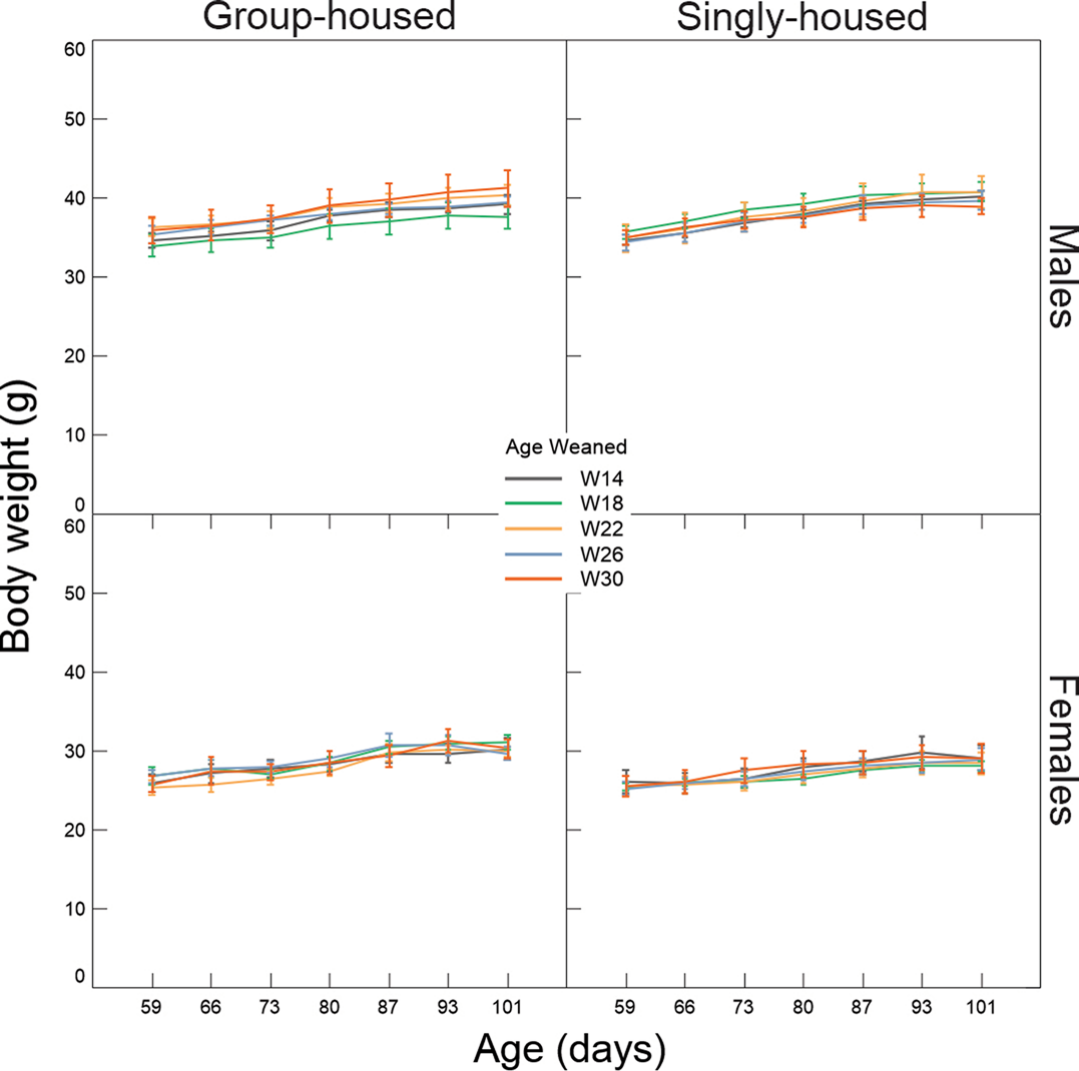
Change in body weight (mean ± SE) across time (ages 59–101) and in relation to age of weaning and housing condition.

During the period of differential housing conditions between 59 to 101 days of age, body weight continued to increase in both sexes; however, body weight did neither vary by weaning age, housing condition, as an interaction between these two or variables, nor as an interaction with age (Fig. 6, Table 2).

### Anxiety in the elevated plus-maze, open field, and social interaction tests

**Figure 7:**
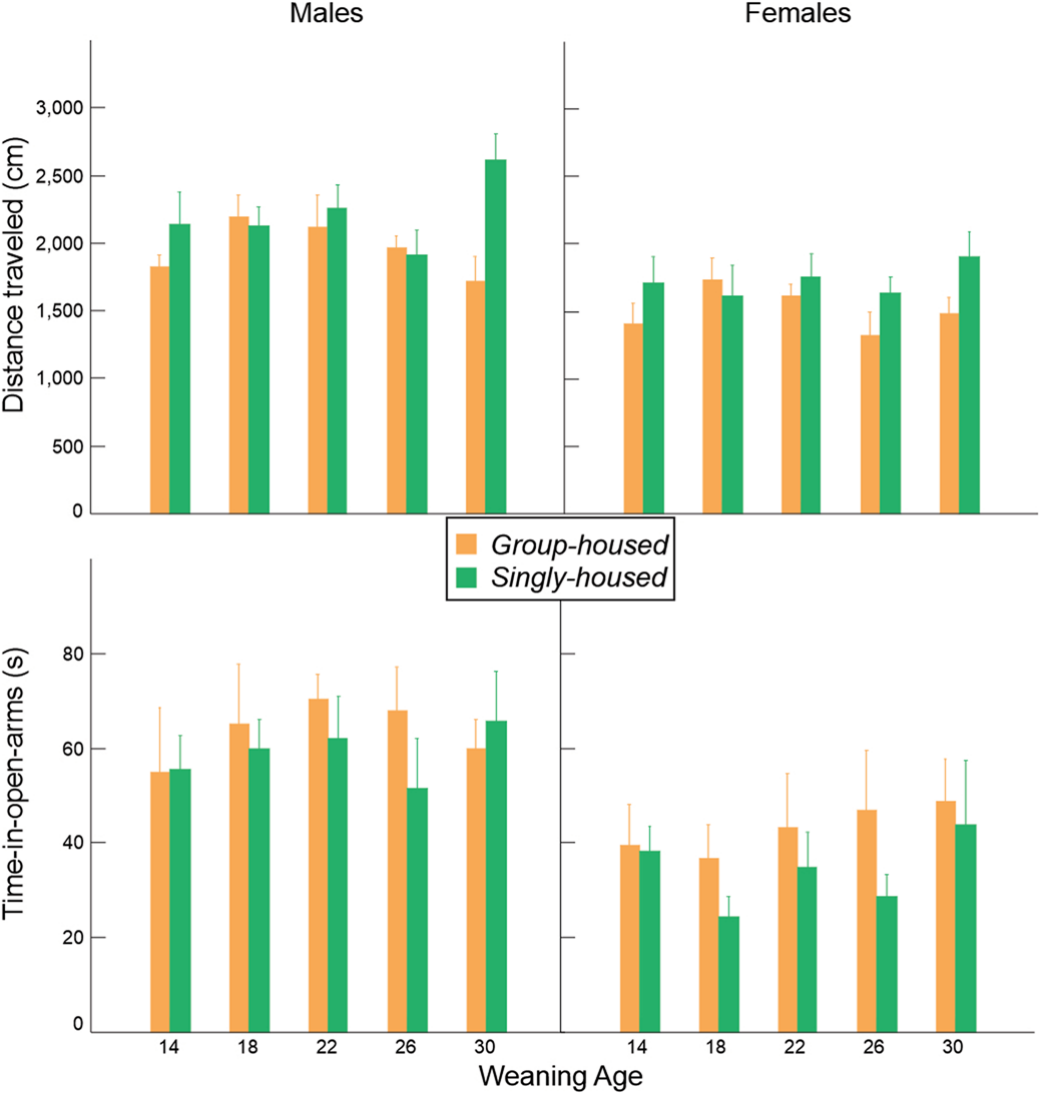
Variation in distance traveled and time-in-open arms (mean ± SE) in relation to weaning age and housing condition in the elevated-plus maze.

Total distance travelled in the elevated-plus maze did not significantly vary with housing condition in both sexes after correction for multiple testing. However, we found a trend in both sexes and after calculating a combined probability we do find an indication that, overall, singly housed mice travelled greater distances than group housed mice (males and females combined: Χ^2^ = 16.094, *p* = 0.003). There is a tentative interaction of weaning age and housing condition in male mice, though after correcting for multiple testing, this effect is no longer statistically significant. For the time-in-open arms, we did not observe any differences (Fig. 7, Table 2).

**Figure 8:**
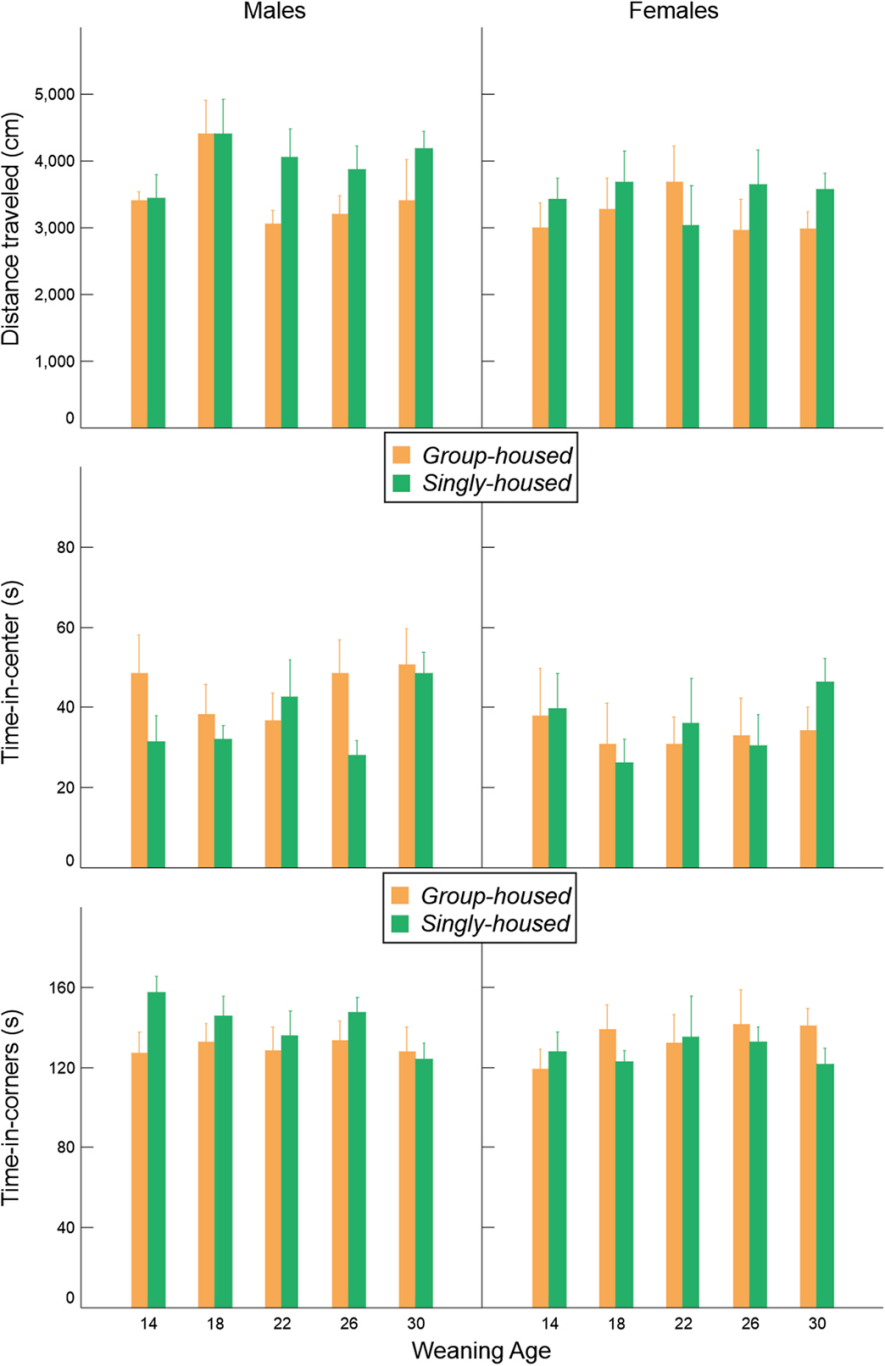
Variation in distance traveled, time-in-center and time-in-corners (mean ± SE) in relation to weaning age and housing condition in the open field.

Behavior in the open field did not vary due to weaning age or housing condition after correction for multiple testing (Fig. 8, Table 2).

**Figure 9:**
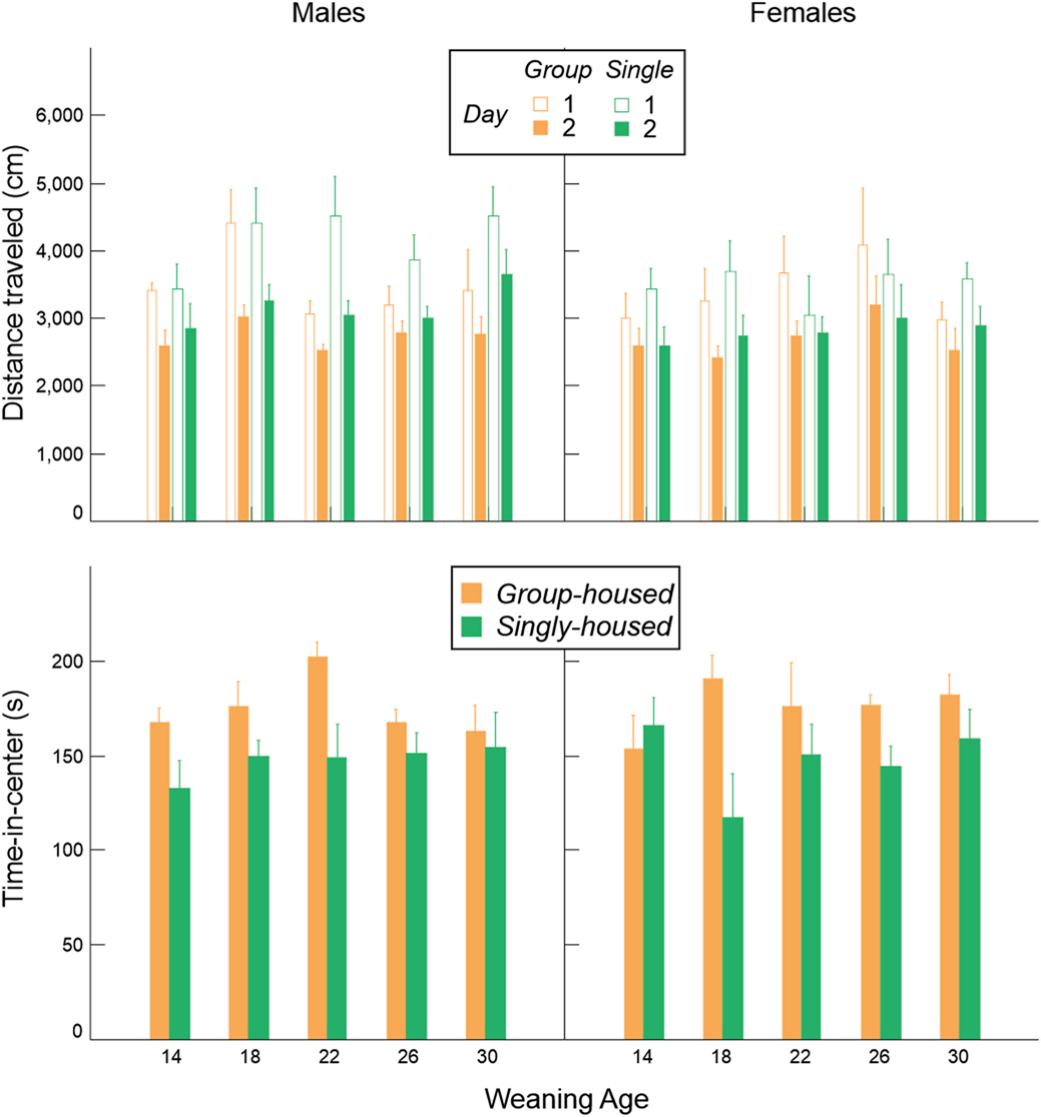
Variation in change in distance traveled (mean ± SE) across two days of testing and time-in-center (i.e., in proximity to the odor stimulus) in relation to weaning age and housing condition in the social odor test.

Behavior in the social odor test varied by housing condition and day of testing, but not as an interaction between any of these variables. Regardless of sex, singly housed mice spent less time in proximity to the social odor cue (time-in-center) and males traveled a greater distance in the open field arena. Additionally, all mice displayed a classic habituation response to the open field arena, traveling smaller distances when exposed to the arena for a second time (Fig. 9, Table 2).

### Adrenocortical activity: glucocorticoid metabolites in feces

**Figure 10:**
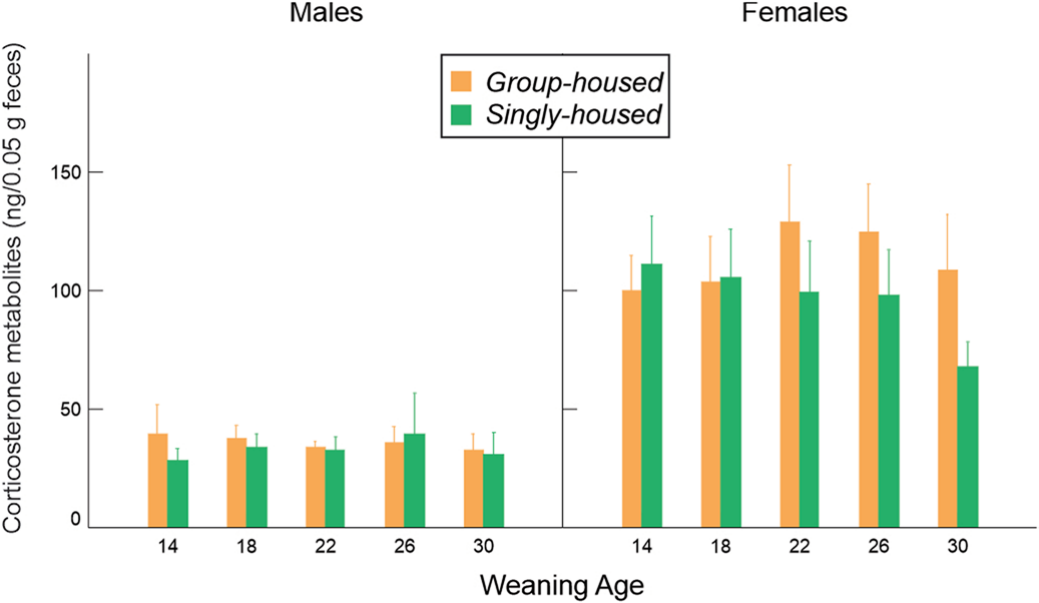
Variation in glucocorticoid metabolite concentrations (mean ± SE) in relation to weaning age and housing condition.

Glucocorticoid levels did not vary with weaning age or housing condition (Fig. 10, Table 2).

## Discussion

To study the effects of variation in weaning age and housing condition on phenotypic differences in mice, we weaned SWISS mice at five different weaning ages, ranging from 14–30 days of age, into same-sex littermate groups of four, and split them into single housing and group housing cohorts at the age of 7 weeks. Both weaning age and housing condition had surprisingly small effects on the animals’ phenotype.

Directly after weaning, we found an effect of weaning age on body weight that was, however, short-lived and from 26 days of age onwards—neither weaning age nor housing condition or a combination of the two had any consistent effects on body weight. As predicted, levels of stereotypic behavior were increased by single housing in both male and female mice, but contrary to our predictions were unaffected be weaning age. Compared to group housed animals, singly housed animals also travelled a greater distance in the elevated plus-maze and in the open field. Additionally, singly housed animals spent less time near the odor stimulus in the social odor test. In contrast, weaning age did not affect behavior in the elevated plus-maze or in the open field—neither in group housed nor in singly housed mice. Only in the social odor test did we find an effect of weaning age on the distance travelled, but only in males. Finally, glucocorticoid metabolite concentration as an indicator of stress was affected neither by weaning age, nor by housing condition.

These findings differ substantially from our predictions and are surprising in light of the published literature, which describes persistent effects of early weaning (day 14) compared to standard weaning age (day 21) on several of the outcomes measured here. By far the most consistently reported effect of early weaning in mice relates to differences in behavioral and neuroendocrine measures of anxiety—with early weaned mice being more anxious compared to standard weaned mice^18,31,59,60^. Similar to the present study, other studies have not reported persistent changes in body weight when comparing early to standard weaned animals^18,31,33,34^. While the effects of early weaning compared to standard weaning are reasonably well described, far fewer studies have compared the effects of late weaning (up to day 35) to standard weaning^27–29^. Mice weaned on day 28 or 35 were behaviorally less anxious in the open-field and elevated plus maze, with concordantly lower levels of corticosterone secretion, and displayed less social behavior in social interaction tests^27–29^.

In contrast to the effects of weaning age, our results on the effects of single housing compared to group housing are reasonably consistent with other studies^35,61–68^. Overall, there seem to be no strong and consistent effects of single housing on measures of anxiety in the open-field and elevated plus maze, on social behavior, and on neuroendocrine responses, and similar to the present study, reported effects are partly sex-specific^40^.

Taken together, our results corroborate the finding that the effects of weaning age and/or single housing may vary due to heterogeneity in conditions and procedures between replicate studies. For example, in a review on the effects of individual housing, Krohn and colleagues highlighted that the method of individual housing, the strain and sex of the mice, cage sizes, flooring conditions, outcomes measured, and data analysis all varied between studies, and attributed differences in the results to such differences in study protocols^40^.

Although similar variation in results exist among studies on early weaning, these have not been discussed in terms of differences in conditions and procedures between studies^30,69^. For example, in their initial work in 2006, Kikusui et al. reported that corticosterone levels were increased for as much as 8 weeks after early weaning, while in 2009 they found a much more limited increase for only 48 h after weaning^18,36^.

In the present study, several procedural aspects may have contributed to the observed results. For example, dams were shipped from the breeder to our laboratory during the second week of pregnancy and had to adapt to a new environment, while in other studies the animals were bred in-house^17,31,59,70^. Potentially, gestational stress due to transport and acclimatization to a new environment may have overridden the effects of early weaning and single housing^71,72^. At weaning, we provided moistened food twice daily to facilitate the transition to feeding from solid food, and 10 g of nesting material to attenuate thermal stress, while other studies weaned the pups into standard laboratory cages without any supporting measures^17,18,36^. Thus, the method of early weaning used here may have been less stressful for the pups compared to other studies.

In light of growing concerns over poor reproducibility in animal research, the heterogeneity in both the results and study protocols among replicate studies questions the external validity of results from individual studies. In particular, differences between study protocols in combination with rigorous within-study standardization may cause conflicting findings between replicate studies^4–6,13^. However, the two procedural factors systematically varied here, weaning age and housing conditions, introduced relatively little variation in the results across a broad range of phenotypic measures. This suggests that other factors that normally vary between laboratories (e.g. strain of mouse, microbiome, and experimenter to name but a few) may account for the observed differences between replicate studies. This calls for further research into the sources of variation in the results between replicate studies, as well as effective ways of designing studies to produce robust and reproducible results.

## Supplementary information

Supplementary information.
