# Supplementary material for: Effects of weaning age and housing conditions on phenotypic differences in mice
Source: Sci Rep. 2020 Jul 15;10:11684. doi: 10.1038/s41598-020-68549-3 (PMC7363894; doi:10.1038/s41598-020-68549-3)
Supplement: Supplementary file 1 — Supplementary information. [file 41598_2020_68549_MOESM1_ESM.docx]

**Effects of weaning age and housing conditions on phenotypic differences in mice**

Jeremy D. Bailoo^1,2,3*^, Bernhard Voelkl^1^, Justin Varholick^1,4^, Janja Novak^1^, Eimear Murphy^5^, Marianna Rosso^1^, Rupert Palme^6^, and Hanno Würbel^1*^

*^1^Division of Animal Welfare, University of Bern, CH*

*^2^Texas Tech Health Sciences Center, School of Medicine, Department of Cell Biology & Biochemistry, Lubbock, TX, USA*

*^3^Texas Tech University,* *Department of Civil, Environmental, and Construction Engineering, Lubbock, TX, USA*

*^4^Department of Biology & UF Genetics Institute, University of Florida, Gainesville, USA*

*^5^Department of Behavioural Biology, University of Münster, Münster, Germany*

*^6^Department of Biomedical Sciences, University of Veterinary Medicine Vienna, AT*

**Corresponding authors: hanno.wuerbel@vetsuisse.unibe.ch and jbailooscience@gmail.com*

**Supplementary Information:**

Number of pages: 3

Number of tables: 4

**Supplementary Table 1.** Distribution of litters to treatment stratified by average litter size and sex ratio


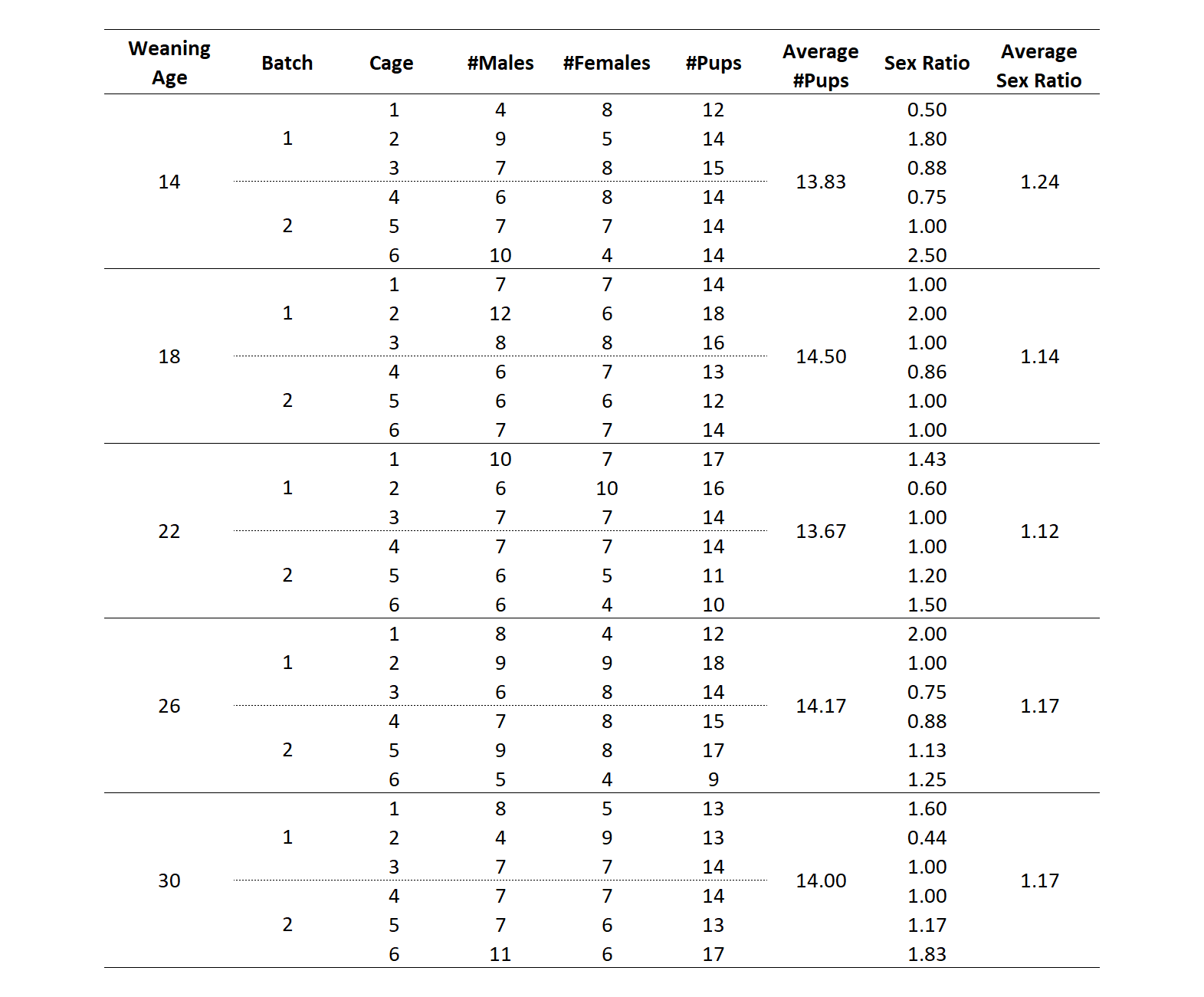


**Supplementary Table 2**. Caging specifications of animals used in this study


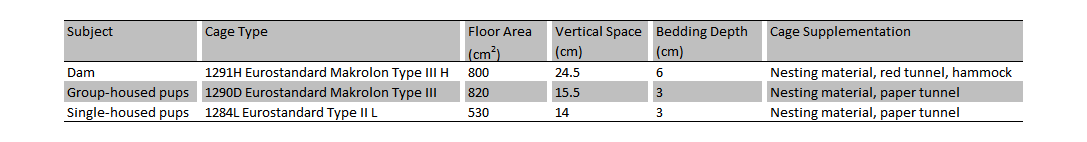


**Supplementary Table 3.** Ethogram for the recording of pup–feeding behavior

**Supplementary Table 4.** Ethogram for the recording of stereotypic behavior. All behaviors coded are mutually exclusive and exhaustive. For stereotypy, the movement/sequence of behavior must be repeated continuously for at least 3s (bar-mouthing) or at least three times in a row without pauses longer than 3s between bouts (circling, twirling, backflipping, route-tracing, jumping).
